# Critical appraisal of Artificial Intelligence and deep-learning tools for intraoperative neurosurgery: hype versus evidence

**DOI:** 10.1097/MS9.0000000000004638

**Published:** 2026-01-21

**Authors:** Tirath Patel, Ehtisham Haider, Amir Riaz, Muhammad Abbas, Bhumi Daishik Patel

**Affiliations:** a Department of Neurosurgery, Trinity Medical Sciences University School of Medicine, Kingstown, Saint Vincent and the Grenadines; b Department of Medicine, Punjab Medical College, Faisalabad, Pakistan; c Department of Medicine, Ayub Medical College, Abbottabad, Pakistan; d Department of Medicine, Windsor University School of Medicine, Cayon, Saint Kitts and Nevis

**Keywords:** clinical validation, intraoperative artificial intelligence, machine learning, neurosurgery, regulatory frameworks

## Abstract

Artificial intelligence (AI) is beginning to aid in several components of the intraoperative workflow, including navigation, instrument tracking, ultrasound analysis, video segmentation, and MRI reconstruction. Early studies demonstrate technical promise but are based on small, single-center datasets with limited heterogeneity and generalizability, and often lead to model overfitting. External validation is rare, and few trials measure the impact on decision-making, complications, or the extent of resection. Differences in annotation standards, imaging protocols and reporting also contribute to the slow speed of translation. Physical, ethical, and regulatory barriers add complexity, especially in settings with limited resources. Recent FDA, EU, and WHO guidance emphasizes lifecycle monitoring, transparency, and real-world evidence, raising the bar for clinical adoption. Progress will require shared databases, standardized reporting, and multicenter implementation studies that track workflow and patient outcomes. Intraoperative AI will definitely not replace a surgeon’s judgment, but if carefully developed and rigorously tested, it may provide meaningful clinical value.

*To the Editor,*

Artificial intelligence (AI) is finding its way throughout the intraoperative workflow; recent reviews describe its use in navigation, instrument tracking, operative video segmentation, ultrasound analysis, and intraoperative MRI reconstruction^[1]^. This collection of literature is not only indicative of actual momentum, but it also reveals the gap between technical potential and validated clinical value^[2]^. This manuscript follows the TITAN checklist to promote transparency in AI reporting^[3]^.

Several studies demonstrate what could be possible. Intraoperative ultrasound has been detected using deep learning with good accuracy in animal models and subsequently in pilot human trials. Notably, the ultrasound foreign-body detection study progressed from animal work to a pilot human cohort of only 28 cases, underscoring the limited nature of current intraoperative AI datasets^[4]^. Others have modeled 5-ALA fluorescence on preoperative MRI in gliomas that appear lower grade on imaging to predict intraoperative biology before the microscope is even involved^[5]^. Speed and improvement in intraoperative MRI reconstruction with deep networks have also been reported, which may otherwise be useful for shortening the workflow and reducing delays in the use of iMRI suites^[6]^. Simultaneously, surgical video segmentation is still developing, and algorithms capable of labeling surgical operations, tracking instruments, and detecting tissue planes can aid real-time guidance^[2]^.

Overall, although this is happening, the evidence base is still thin. Most studies use small datasets selected from a single center and exhibit minimal demographic or technical heterogeneity. It is not uncommon to overfit the models, and performance tends to decrease when the environment in which they are tested differs. External validation remains the exception, and future trials assessing the impact on decision making, complication rates, or level of resection are uncommon^[1]^. There are differences in annotation standards, inconsistencies in imaging protocols, and gaps in reporting in inter-study comparisons. Such issues slow the translation process and complicate the task of estimating the algorithm’s behavior in everyday practice.

Physical obstacles are also significant. AI systems typically require specific hardware, advanced imaging, and technical assistance. These are costly requirements that are often not viable in low- and middle-income countries, where neurosurgical capacity is already strained, and any useful tool must be robust and inexpensive to repair and sustain. Among the ethical and regulatory concerns are unclear model behavior, inadequate reporting of failure modes, and liability issues arising from automated suggestions that affect real-time surgical decision-making^[2]^. There is little research on human factors, despite the interpretation and action of teams based on AI output being a potential safety factor as much as the model’s actual performance.

The need for more rigorous evidence is only more urgent due to recent regulatory changes. The FDA’s Software as a Medical Device guidance on AI/ML systems highlights the need to monitor the life cycle and conduct a clear, risk-based review^[7]^. Intraoperative AI is considered high-risk under the EU AI Act (2024), which requires strict audit trails, documented risk management, and human oversight. The WHO 2024 guide on AI in health systems recommends continuous real-world evidence after market launch and requires clear accountability models. The common theme across these documents is that AI tools used in surgery should meet high evidentiary standards.

A way out is required. External datasets, standardized reporting, and intraoperative AI assessment should be the minimum validation requirements. Imaging, operative, and patient outcome data, as well as AI outputs, would be collected on a multicenter basis, enabling real-world benchmarking and model drift identification. The implementation studies would need to monitor changes in workflow, communication, decision-making, and patient outcomes resulting from AI tools in the long run. Only through long-term postdeployment surveillance can we determine whether these systems are safe and beneficial beyond controlled research.

AI will not substitute intraoperative judgment, but when it is created conscientiously, it can be enhanced. To achieve progress, it requires disciplined approaches, open reporting, and a dedication to test tools that are not only accurate but also clinically useful. The discipline must be open to innovation, but must have rigorous, generalizable, and applicable evidence for the environments where neurosurgical services might be most required. We urge the neurosurgical community and regulatory bodies to prioritize multicenter validation frameworks and post-deployment surveillance to ensure safe and meaningful integration of intraoperative AI.

## Data Availability

No new dataset was generated during the study.

## Research registration unique identifying number (UIN)

Not required because the study is a letter to the editor.
